# Metabolic Dysfunction-Associated Steatotic Liver Disease in Type 2 Diabetes Patients—The Relationship with Platelets Indicators

**DOI:** 10.3390/medicina60122091

**Published:** 2024-12-21

**Authors:** Danusia Onișor, Andrada Larisa Roiban, Simona Cernea

**Affiliations:** 1 Department ME2, Internal Medicine VII, George Emil Palade University of Medicine, Pharmacy, Science and Technology of Târgu Mureş, 540142 Târgu Mureş, Romania; danusia.onisor@umfst.ro; 2 Gastroenterology Clinic, Mureș County Clinical Hospital, 540103 Târgu Mureş, Romania; 3 Diabetes Compartment, Mediaș Municipal Hospital, 551030 Mediaș, Romania; andrada_pasc@yahoo.com; 4 Doctoral School of Medicine and Pharmacy, George Emil Palade University of Medicine, Pharmacy, Science, and Technology of Târgu Mureş, 540142 Târgu Mureş, Romania; 5 Department M3, Internal Medicine I, George Emil Palade University of Medicine, Pharmacy, Science, and Technology of Târgu Mureş, 540142 Târgu Mureş, Romania; 6 Diabetes, Nutrition and Metabolic Diseases Outpatient Unit, Emergency County Clinical Hospital, 540136 Târgu Mureş, Romania

**Keywords:** platelets, metabolic dysfunction-associated steatotic liver disease, type 2 diabetes mellitus

## Abstract

*Background and Objectives*: Metabolic dysfunction-associated steatotic liver disease (MASLD) is an important chronic liver disease with major health risks, especially in the presence of T2DM, but the pathophysiology of this condition is not fully understood. This study aimed to investigate the platelet hematometric indices in patients with T2DM and MASLD. *Materials and Methods*: Demographic and medical (including anthropometric) data were collected from 271 participants, from whom blood samples were also drawn in fasting conditions for complete blood count, liver and metabolic panel, ferritin, haptoglobin, creatinine, and fibrosis markers. The correlations of main platelet parameters with clinical and laboratory data were investigated by bivariate and multiple regression analyses. *Results*: The median platelets number was 235·10^3^/μL, and thus, the study population was divided into two subgroups: with higher and lower numbers (group 1 (mean): 286.38 ± 43.29·10^3^/μL and group 2 (mean): 188.12 ± 39.77·10^3^/μL). Despite similar BMIs, group 2 had higher fatty liver index (FLI) (84.44 ± 18.04 vs. 79.85 ± 17.98; *p* = 0.0088) and insulin resistance (HOMA-IR: 3.16 ± 1.50 vs. 2.63 ± 1.31; 0.0008), higher direct bilirubin, transaminases, uric acid, and ferritin concentrations. Higher percentages of males and subjects with HOMA-IR values >2.5 were accounted for in this group. In the multiple regression analyses, the platelet count and plateletcrit (PTC) correlated independently with sex, leucocyte count, HOMA-IR, and bilirubin concentrations (*p* < 0.0001). The platelet distribution width (PDW) was positively correlated with insulin resistance in two separate analyses (β = 0.060; *p* = 0.0004, and β = 0.052; *p* = 0.0025), and with GGT, while the mean platelet volume presented a weak but significant positive association with FLI. Patients with higher HOMA-IR had higher PDW and a lower platelet count and PTC. *Conclusions*: Male patients with T2DM and MASLD had lower platelet count and PTC and larger PDW. Higher insulin resistance was associated with lower platelet count and PTC and higher PDW.

## 1. Introduction

Metabolic dysfunction-associated steatotic liver disease (MASLD) has become the most frequent chronic liver disease in the adult population worldwide, with a current prevalence of about 30% [1,2]. MASLD is defined by the presence of hepatic steatosis associated with at least one cardio-metabolic risk factor and includes several potentially progressive liver conditions, from simple steatosis to steatohepatitis with different stages of fibrosis, up to cirrhosis and hepatocellular carcinoma (HCC) [3,4]. Besides other risk factors, obesity, insulin resistance, and type 2 diabetes mellitus (T2DM) are associated with MASLD progression [4,5,6]. In fact, not only diabetes but also early glycemic derangements seem to be associated with more advanced fibrosis [5,7,8]. The identification of biomarkers that detect early stages of the disease, facilitate a better understanding of disease pathogenesis, identify patients at risk of faster disease progression, or constitute a therapeutic target is greatly needed and has been pursued for a long time.

Platelets are the smallest blood cells formed in the hematogenous marrow by the fragmentation of megakaryocytes [9]. They are mainly involved in thrombosis and hemostasis but also inflammation, immune responses, angiogenesis, fibrosis, tissue regeneration, etc. [10,11,12,13,14]. The liver plays a significant role in the function and number of platelets, and in the context of liver fibrosis, they can have both pro- and anti-fibrotic effects [13]. The liver contributes to the production of platelets through the synthesis of thrombopoietin, which decreases advanced chronic liver disease, resulting in thrombocytopenia [15,16]. In addition, thrombocytopenia may also result from bone marrow suppression, the splenic sequestration of platelets, or their increased destruction, which usually occurs in more advanced stages of liver disease (cirrhosis) [16]. On the other hand, it has been suggested that activated platelets might play a role in liver regeneration through the release of vascular endothelial growth factor (VEGF) that stimulates the endothelium cells to produce hepatocyte growth factor (HGF) and interleukin 6 [17].

Patients with MASLD seem to have higher mean platelet volume (MPV) compared to healthy subjects, and the same was shown in subjects with diabetes and impaired fasting glucose [18,19]. Some data suggest that the activation of platelets is associated with the increase in their volume, and glycemic variability, insulin resistance, and inflammation are important determinants [12,20,21,22]. In fact, increased platelet reactivity seen in patients with T2DM is considered a pro-thrombotic state and is likely to be involved in the chronic vascular complications of diabetes [23]. The mechanisms behind these correlations are not entirely clear, but apparently, hyperglycemia increases platelet reactivity through induction of non-enzymatic glycation of surface platelet proteins, osmotic effect, or activation of protein kinase C [22]. In addition, both insulin resistance and deficiency, hyperlipidemia, oxidative stress, endothelial dysfunction, inflammation, and enhanced thromboxane biosynthesis play an important role [22,24]. Chronic inflammation plays an important role during MASLD progression, as the recruitment and activation of diverse innate and adaptive immune cell populations, activation of the NOD-, LRR- and pyrin domain-containing protein 3 (NLRP3) inflammasome and release of pro-inflammatory mediators lead to hepatocytes death and liver fibrosis [25,26,27]. It is also a key driver of T2DM onset and progression, as well as for associated disorders [28]. In this context, platelets are involved in inflammatory processes, innate and adaptive immune responses, and the development of MASH [26,29]. Moreover, platelet parameters have been shown to be modified in other inflammatory conditions [30].

The aim of this study was to investigate the platelet hematometric indices in patients with T2DM and MASLD since there are limited data in the literature regarding these associations.

## 2. Materials and Methods

Study population. A detailed description of the material and methods, and inclusion and exclusion criteria used in this study has been published elsewhere [31]. Briefly, starting in July 2022 for a 12-month duration, we included male and female patients with T2DM and MASLD, recruited mostly from the Diabetes, Nutrition and Metabolic Diseases Outpatient Unit of the Emergency County Clinical Hospital of Târgu Mureș and from the Gastroenterology Department of the County Clinical Hospital of Târgu Mureș. Patients were enrolled if they were ≥30 years old and had T2DM and non-alcoholic fatty liver disease (NAFLD) by patient history and hepatic ultrasound. We used the NAFLD definition (hepatic steatosis/steatohepatitis in the absence of other secondary causes of liver disease) as an inclusion criterion, but in June 2023, the change in terminology and definition to MASLD was proposed and later largely endorsed [32]. Since all our patients fulfilled the MASLD definition (had liver steatosis confirmed by ultrasound and at least one additional criterion (T2DM)), we have, therefore, adopted the new term MASLD to characterize this study population.

Clinical and laboratory evaluations. We collected demographic and medical data (including information about patients’ lifestyles, anthropometric parameters, heart rate, and blood pressure). The alcohol intake was specifically assessed by the AUDIT-C test and an additional questionnaire with secondary interview. The height, weight, waist and hip circumferences, and blood pressure were measured by standard methods. The body mass index (BMI) was calculated by dividing the weight by height^2^ (kg/m^2^). Liver ultrasound (US) was employed to confirm and evaluate liver steatosis by using a Hitachi Arietta v70 US machine [33]. US B-mode imaging allowed the estimation of the degree of fatty infiltration in the liver. The quantification of hepatic steatosis was obtained through the subjective assessment of the brightness of the liver (the contrast between the liver and the kidneys), the appearance of the intrahepatic vessels, the liver parenchyma, and the diaphragm [33].

Blood samples were collected in fasting conditions, and the complete blood count was analyzed shortly afterward on a five-part differential automated hematology BC6200 analyzer (Mindray, India). Serum aliquots were stored at −80 °C for subsequent analysis of a number of parameters: metabolic panel (blood glucose, glycated hemoglobin (HbA1c), HDL (high-density lipoprotein) cholesterol, LDL (low-density lipoprotein) cholesterol and total cholesterol, triglycerides, uric acid, C-peptide), liver panel (aspartate aminotransferase (ASAT), alanine aminotransferase (ALAT), gamma-glutamyl transpeptidase (GGT), direct bilirubin, albumin), ferritin, haptoglobin, creatinine, and fibrosis markers (tissue inhibitors of metalloproteinase-1 (TIMP-1), procollagen III N-terminal propeptide (PIIINP), and cytokeratin 18 (CK-18)). The biochemical parameters were analyzed on a Cobas Integra 400plus (Roche Diagnostic, Mannheim, Germany). Albumin, haptoglobin, and HbA1c were measured by an immunoturbidimetric method, while the uric acid, liver enzymes, bilirubin, creatinine, glucose, and lipids were measured by a spectrophotometric method. The C-peptide, ferritin, and SHBP were analyzed on Immulite 2000 XPI system (Siemens, Munich, Germany) by a solid phase, two-site chemiluminescent immunometric assay. The fibrosis markers were assessed by ELISA method: TIMP-1 (ABclonal, Lonsee, Germany; detection range: 62.5–4000 pg/mL; intra-assay coefficient of variation (CV) < 10%), PIIINP (ABclonal; detection range: 0.312–20 ng/mL; intra-assay CV ≤ 5.5%), CK-18 (ABclonal; detection range: 62.5–4000 pg/mL; intra-assay CV < 10%).

The Homeostatic Model Assessment (HOMA) for Insulin Resistance (HOMA-IR) was calculated by using the HOMA calculator version 2.2.3 [34]. The Fatty liver index (FLI) was calculated with the following formula: FLI = (e^0.953 × loge (TG) + 0.139 × BMI + 0.718 × loge (GGT) + 0.053 × waist − 15.745^)/(1 + e^0.953 × loge (TG) + 0.139 × BMI + 0.718 × loge (GGT) + 0.053 × waist − 15.745^) × 100 [35].

Statistical analysis. Normality of data was verified with the Kolmogorov–Smirnov test. The comparison of continuous variables was performed by using the Student’s *t*-test (for normally distributed variables) or Mann–Whitney test (for non-parametrical variables), while the chi-squared test was employed for analysis of categorical variables. The relationship between two variables of interest was investigated by Spearman’s or Pearson’s test, with data presented as correlation coefficients r [95% CI (confidence interval)]. Multiple regression analyses were employed for the analysis of more than two variables to test the independent association between platelet indices and other parameters of interest. Statistical analysis was performed using GraphPad InStat 3. The statistical significance was assigned at *p* value < 0.05.

## 3. Results

In this analysis, data from 271 T2DM patients with MASLD were included (149 females and 122 males), with a mean age of 65.0 ± 8.4 years and a mean duration of diabetes of 9.7 ± 5.0 years. The median value of the platelets number was 235·10^3^/μL (one outlier excluded), and thus, the study population was divided into two subgroups: group 1—with higher platelet count (>235·10^3^/μL) and group 2—with lower platelet count (≤ 235·10^3^/μL). There were no differences in age (64.45 ± 8.46 vs. 65.46 ± 8.26 years, *p* > 0.05), duration of diabetes (9.53 ± 4.71 vs. 9.82 ± 5.19 years, *p* > 0.05), or BMI (33.55 ± 5.08 vs. 34.63 ± 5.48 kg/m^2^, *p* > 0.05) between the two subgroups, but the sex distribution was different: the lower platelet number group had higher percentage of males (53.96%), while group 1 had higher percentage of women (64.39%), *p* = 0.0033. Of all patients, 98.2% received metformin, 24.0% a sodium–glucose cotransporter-2 (SGLT-2) inhibitor, 34.7% a glucagon-like peptide-1 (GLP-1) receptor agonist, 7.4% a dipeptidyl peptidase 4 (DPP4) inhibitor, 11.1% a sulphonilyurea, and 24.35% insulin, without any difference between subgroups (*p* > 0.05 for all).

The mean platelet count in group 1 was 286.38 ± 43.29·10^3^/μL, while in group 2, it was 188.12 ± 39.77·10^3^/μL (*p* < 0.0001). Table 1 presents the hematological and biochemical data of the two groups. T2DM-MASLD patients with lower platelet numbers (group 2) also had a lower number of leucocytes, neutrophils, lymphocytes, monocytes, and mean plateletcrit (PTC), and higher mean corpuscular volume (MCV) and mean corpuscular hemoglobin (MCH) compared with the other group (Table 1a). Additionally, the lower platelet count group had increased MPV and platelet distribution width (PDW) (Table 1a).

**Figure 1:**
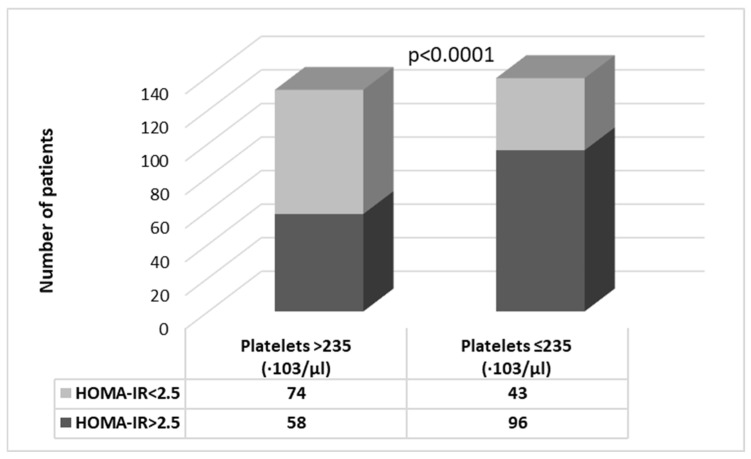
Number of patients with significant insulin resistance (HOMA-IR > 2.5) in each study subgroup divided by mean number of platelets (>235·10^3^/μL and ≤235·10^3^/μL, respectively).

Despite having similar BMIs, the lower platelet count group had higher FLI (84.44 ± 18.04 vs. 79.85 ± 17.98; *p* = 0.0088) and higher insulin resistance (HOMA-IR: 3.16 ± 1.50 vs. 2.63 ± 1.31; 0.0008). More patients had HOMA-IR values >2.5 (indicative of insulin resistance) in this group compared with group 1 (69.1% vs. 43.9%; *p* < 0.0001) (Figure 1). In fact, the group with lower platelet count also presented higher transaminase values, as well as bilirubin, uric acid, ferritin, and C peptide concentrations, but lower haptoglobin levels (Table 1b). The creatinine concentrations were lower in group 1 (Table 1b), but the eGFR values were similar (87.81 ± 15.42 vs. 84.03 ± 18.48 mL/min/1.73 m^2^; *p* = 0.1827). For 264 subjects, the TIMP-1, CK-18, and PIIINP concentrations were also measured, with no significant differences between the two groups for TIMP-1 (3.81 ± 0.83 vs. 3.85 ± 0.70 ng/mL, *p* = 0.9018), and CK-18 (71.61 ± 119.16 vs. 62.59 ± 64.01 pg/mL; *p* = 0.4737), while the PIIINP levels were slightly higher in group 1 (28.56 ± 17.04 vs. 27.70 ± 38.69 ng/mL; *p* = 0.0006).

### Correlations Between Platelet Indices and Laboratory and Clinical Data

**Figure 2:**
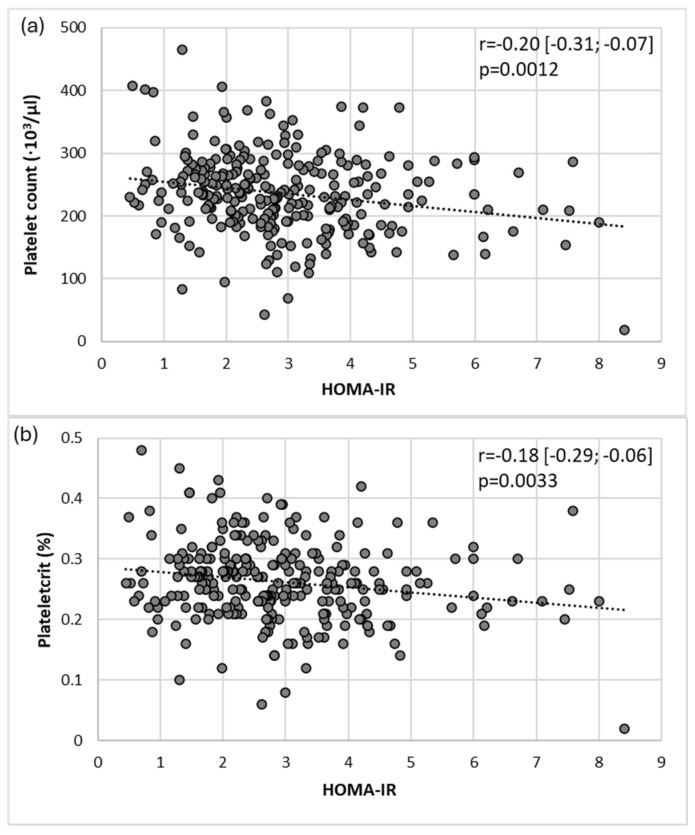
Associations between HOMA-IR and (**a**) platelet count and (**b**) PTC in MASLD-T2DM patients.

The correlations between main platelet parameters and other clinical and laboratory data were investigated by bivariate and multiple regression analyses. The main results of the bivariate analyses for platelet numbers, PTC, and PDW are presented in Table 2 and Figure 2. There were positive associations between the platelet count and PTC with white blood cell counts, haptoglobin, and PIIINP, as well as negative associations with red blood cell indices, sex, insulin resistance, and liver markers (Table 2). The MPV was associated positively only with BMI (r = 0.16 [0.04; 0.28], *p* = 0.007) and FLI (r = 0.15 [0.03; 0.27]; *p* = 0.0125) and negatively with MCHC (r = −0.15 [−0.26; −0.02]; *p* = 0.0163) and diabetes duration (r = −0.15 [−0.27; −0.03], *p* = 0.0153). Age was only correlated with PDW (r = −0.15 [−0.27; −0.03], *p* = 0.0134), and so was smoking (r = 0.14 [0.01; 0.26]; *p* = 0.0247), but PDW also correlated positively with red blood cells indices, insulin resistance, and metabolic and liver parameters (Table 2). For other clinical and laboratory parameters there were no significant correlations observed in the bivariate analyses with neither platelet indices.

In the multiple regression analyses, the platelet count correlated independently with sex, leucocyte count, HOMA-IR, and bilirubin concentrations (R^2^ = 33.39%; *p* < 0.0001) (but not with HEM, PIIINP, alcohol intake, ferritin, haptoglobin, ASAT, ALAT, or GGT) (Table 3). Similar results were obtained when PTC was analyzed as the independent variable (Table 3). For the MPV, the correlations were not quite statistically significant (R^2^ = 8.26%; *p* = 0.0578), while PDW correlated with sex (male subjects had higher PDW), HOMA-IR, alcohol intake, and GGT (Table 3).

Two additional multiple regression analyses were performed to better elucidate the associations between platelet indices (MPV and PDW) and the parameters of interest by including only those with significant correlations observed in the bivariate analyses. For MPV, the analysis included diabetes duration, MCHC, and FLI (since BMI was already incorporated in the formula), and the results indicated a weak but significant association of MPV with FLI (β = 0.009 [SE = 0.004]; *p* = 0.0302) (R^2^ = 4.28%; *p* = 0.0086). The second one comprised the parameters significantly associated with PDW in the bivariate analysis (sex, HOMA-IR, HGB, ferritin, ASAT, ALAT, GGT, triglycerides, HDL cholesterol, creatinine, PIIINP, age, and smoking) (R^2^ = 15.98%; *p* < 0.0001) and showed that insulin resistance (β = 0.052 [SE = 0.017]; *p* = 0.0025), GGT (β = 0.001 [SE = 0.001]; *p* = 0.0194), and ferritin (β = −0.0004 [SE = 0.0002]; *p* = 0.0374) correlated with PDW.

**Figure 3:**
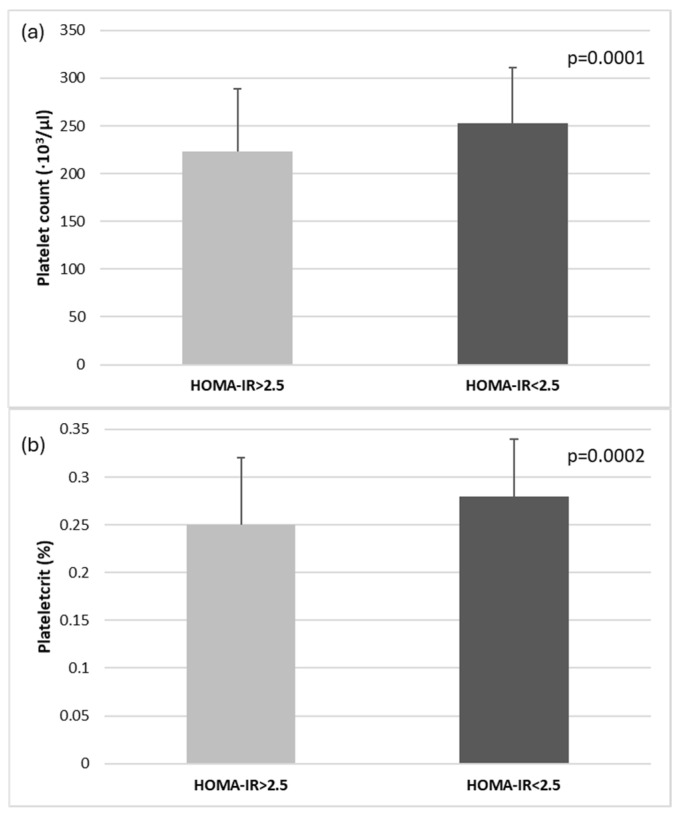
(**a**) Platelet count values based on insulin resistance status (HOMA-IR values below or above 2.5) and (**b**) PTC based on insulin resistance status (HOMA-IR values below or above 2.5).

Since insulin resistance appeared as a significant biomarker correlated with main platelets indices, a supplementary analysis based on HOMA-IR values (insulin resistance status) confirmed higher PDW values (16.27 ± 0.40 vs. 16.12 ± 0.28, *p* = 0.0004), and lower platelet count and PTC in T2DM-MASLD patients with HOMA-IR > 2.5 (Figure 3).

In addition, patients with direct bilirubin levels ≥ 0.3 mg/dL had lower platelet counts and PTC compared with those with bilirubin concentrations < 0.3 mg/dL (207.40 ± 59.83 vs. 241.45 ± 63.86 mg/dL, *p* = 0.0015, and 0.23 ± 0.07 vs. 0.27 ± 0.06%, *p* = 0.0034, respectively).

## 4. Discussion

MASLD emerges as the current main cause of advanced liver disease, and accumulating evidence implies that platelets are involved in the pathogenesis of MASLD through proinflammatory and profibrotic effects [36]. It has been suggested that the accumulation of platelets in the liver and their activation might be caused at least in part by lipids (lipid toxicity) and that they can further attract other immune cells like leukocytes, release factors that prime an immunoinflammatory reaction, activate the stellate cells, and interact with the endothelium in the liver [36]. Inflammation is, in fact, an important mechanism that favors the progression of MASLD towards more advanced stages (steatohepatitis) [37].

Several papers have evaluated the relationship between platelets and hepatic steatosis/fibrosis with mixed results, but to the best of our knowledge, our research is the first that investigated this association in subjects with T2DM and MASLD. The evidence so far is not consistent with regards to the relationship between platelet count and liver steatosis [38,39,40]. However, the meta-analysis of 19 studies (3592 NAFLD patients and 1194 healthy controls) by Li L et al. demonstrated that the platelet count was lower in patients with NAFLD versus control subjects (SMD = −0.66 [95%CI:−1.22;−0.09], *p* = 0.023) and in those with non-alcoholic steatohepatitis (NASH) (SMD = −0.86 [95%CI:−1.20;−0.52], *p* < 0.001) [41]. In addition, two studies demonstrated lower platelet count with increasing liver fibrosis severity [42,43]. Our research showed that the platelet number correlated negatively with markers of liver steatosis (FLI) and liver function tests (transaminases, GGT, bilirubin), suggesting that the platelet count decreases as the liver disease progresses. This is in accordance with the study by Liu F et al. that demonstrated a significant (although small) decrease in platelet count after 5 years of follow-up in NAFLD patients (from 220.6  ±  42.22 (10^9^/L) to 208.41  ±  40.70 (10^9^/L), *p* <  0.0001) [44]. In addition, we have found that the MPV was correlated weakly but significantly with FLI, indicating a correlation with liver steatosis. This independent association was, in fact, confirmed in two previous studies that included obese subjects with NAFLD [45,46]. The study by Ozhan K et al. reported that NAFLD was an independent predictor of MPV (Odds Ratio: 21.98; *p* = 0.006]. On the other hand, Malehmir M. and colleagues have reported a significant increase in intrahepatic platelet numbers in several murine NASH models (but not in simple steatosis) with increased platelet aggregation and activation, and the same was demonstrated in NAFLD/NASH patients [47]. Additionally, they showed a decrease in intrahepatic platelet accumulation and limited hepatic immune-cell trafficking after antiplatelet therapy, suggesting a pivotal role of platelet trafficking and activation in the liver for NASH progression [47]. Nevertheless, more research is needed to elucidate the dynamics of circulating intrahepatic platelet count changes as the disease advances.

Our data showed an independent correlation between the platelet count and direct bilirubin concentrations. Patients with direct bilirubin levels ≥0.3 mg/dL had lower platelet count and PTC compared with those with lower bilirubin levels (although the mean value was still in the normal range). A moderate negative correlation was found between platelet number and direct bilirubin (and the same was true for PTC). In our opinion, this is rather an indication of subtle changes in platelet parameters that occur as the liver disease progresses. Conjugated hyperbilirubinemia is seen in hepatocellular injury, intrahepatic cholestasis, or other conditions [48]. In a retrospective study that included 223 patients with thrombocytopenia, Hancox et al. showed a negative correlation between serum bilirubin and platelet count as an indicator of liver dysfunction [49]. Interestingly, the study by Pennell et al. demonstrated the anti-platelet effects of a conjugated bilirubin analog following acute ex vivo exposure [50].

The study by Panke et al., which included 441 patients with liver-biopsy-diagnosed NAFLD without cirrhosis, showed that thrombocytopenia was associated with male sex and hemoglobin levels [51]. Our data also indicated that the male sex was independently associated with lower platelet count and PTC (but higher PDW), and the sex distribution was different in the two study subgroups: there were more male patients in the lower platelet number group (53.96%) and more females in the higher platelet number group (64.39%). Some studies in the general population have indicated sex- and age-based differences in platelet number and characteristics, with males and older age being associated with lower platelet numbers [52,53,54]. The reasons behind the platelet count and platelet feature variability with sex are not entirely clear, but sex-based differences in the concentration of some platelet-derived growth factors (such as platelet-derived growth factor) have been described [54,55]. Moreover, it appears that the platelet count variability, even within the normal range, contributes to a higher risk of morbidity (cancer, cardiovascular disease, etc.) and mortality [56]. Therefore, sex-adjusted normal ranges for platelet count, as well as for liver fibrosis markers that account for platelet number (such as FIB4), should probably be proposed to better reflect these differences.

Platelets have insulin receptors, and insulin modulates their activity [57]. The activation of platelets is associated with the increase in their volume and PDW [58]. In insulin-resistant states, the inhibitory effect of insulin on platelets is compromised [58]. Some (but not all) studies have suggested that MPV is increased in insulin-resistant conditions [59,60]. Our data did not indicate a direct correlation of MPV with insulin resistance; instead, it correlated with the BMI (and FLI), and this might be explained by more adipokines/growth factors being released in the circulation by the adipose tissue that stimulates the bone marrow to produce larger and more reactive platelets [58,61]. HOMA-IR correlated positively with PDW in our study population. Higher PDW indicates a larger range of platelet sizes, which might result from more activation and destruction of platelets [57]. In accordance with our results, other data in the literature indicated higher PDW in insulin-resistant states (such as T2DM) and in NAFLD patients [41,62]. The results also showed a negative correlation between HOMA-IR with platelet number and PTC. In contrast, the study by Park et al., which included 1133 Korean adolescents, demonstrated a positive association between insulin resistance and platelet count, with higher HOMA-IR values in higher platelet count quartiles [63]. The differences might be due to differences in study populations. It is known that the metabolic dysregulation resulting from insulin resistance has a significant contribution to the progression to steatohepatitis, and more advanced MASLD/fibrosis is associated with lower platelets number, possibly related, at least in part, to the degree of fatty liver infiltration [6,64]. However, this hypothesis needs further investigation. In this regard, the study by Michalak et al. demonstrated a strong negative correlation between PTC and NAFLD-related liver fibrosis [65].

Our study has several limitations. First, the diagnosis of hepatic steatosis was based on ultrasonography and non-invasive biomarkers, as more advanced techniques (magnetic resonance imaging (MRI)-based) were not available, and liver biopsy was not performed. Secondly, this was a cross-sectional study, and the observation of changes in platelet number/characteristics over time as MASLD progresses may provide more valuable information. Thirdly, the number of patients with thrombocytopenia/thrombocytosis was low, and most platelet-related indices were within normal values, so we could only observe fine changes associated with it. A larger cohort size would also allow subgroup analysis and provide more generalizable results. Finally, the cross-sectional design of the study limits causal inferences regarding the relationship between platelet indices and MASLD progression. A long-term follow-up of the cohort would clarify whether changes in platelet indices predict MASLD progression (worsening of hepatic steatosis and/or fibrosis) or its regression. This would also allow the observation of any correlation between changes in platelet parameters and T2DM outcomes, mainly cardiovascular (which, in fact, are associated with both conditions). Ideally, long-term monitoring should be performed in a liver-biopsy-proven MASLD cohort, which would not only offer the opportunity for a more accurate evaluation of MASLD progression but also for the study of intra-hepatic platelets in conjunction with changes of peripheral platelets. Nevertheless, these observations might open future perspectives for studies focusing on the dynamics of platelets and their role in the pathogenesis and progression of MASLD in patients with and without T2DM. The monitoring of platelet indices might serve as biomarkers of disease progression/severity. They might also be indicators of significant insulin resistance, as this study indirectly confirmed that platelets are a site of insulin resistance.

## 5. Conclusions

Male T2DM patients with MASLD had lower platelet count and PTC and larger PDW. Higher insulin resistance is independently associated with lower platelet count and PTC and higher PDW. The MPV was weakly correlated with FLI.

## Figures and Tables

**Table 1:** Hematologic and biochemical data in the two study subgroups (MCV = mean corpuscular volume; MCH = mean corpuscular hemoglobin; MCHC = mean corpuscular hemoglobin concentration; MPV = mean platelet volume; PDW = platelet distribution width; ASAT = aspartate aminotransferase; ALAT = alanine aminotransferase; GGT = gamma glutamyl transpeptidase; HbA1c = glycated hemoglobin; results are presented as mean ± SD for normally distributed data, and median (min-max) for non-parametrical data).

	Group 1 (Platelet Number > 235·10^3^/μL) (n = 132)	Group 2 (Platelet Number ≤ 235·10^3^/μL) (n = 139)	*p*
(**a**) Hematological data			
Red blood cell count (10^6^/μL)	4.81 ± 0.48	4.80 ± 0.54	0.9071
Hemoglobin (g/dL)	14.05 (11.30–17.90)	14.39 ± 1.74	0.0680
Hematocrit (%)	42.83 ± 3.84	43.56 ± 4.99	0.1790
MCV (fL)	89.38 ± 5.62	90.88 ± 5.12	0.0023
MCH (pg)	29.50 (19.60–37.60)	30.20 (18.40–33.90)	0.0021
MCHC (g/dL)	32.90 ± 0.81	33.10 (28.50–34.70)	0.0621
Leucocyte count (10^3^/μL)	8.28 ± 1.88	7.07 ± 1.66	<0.0001
Neutrophil count (10^3^/μL)	5.07 ± 1.51	4.03 (1.54–8.67)	<0.0001
Lymphocyte count (10^3^/μL)	2.42 (1.12–4.68)	2.14 ± 0.62	0.0004
Monocyte count (10^3^/μL)	0.48 (0.27–0.84)	0.43 (0.09–0.90)	0.0116
Eosinophil count (10^3^/μL)	0.18 (0.0–0.89)	0.18 (0.0–1.32)	0.9271
Basophil count (10^3^/μL)	0.04 (0.0–0.11)	0.04 (0.0–0.11)	0.1347
Plateletcrit (%)	0.31 ± 0.05	0.22 ± 0.05	<0.0001
MPV (fL)	10.79 ± 1.04	11.74 ± 1.29	<0.0001
PDW (10GSD)	16.1 (15.4–16.9)	16.3 (15.4–19.0)	<0.0001
(**b**) Biochemistry data			
Fasting blood glucose (mg/dL)	136.55 (87.34–320.11)	137.59 (91.85–326.2)	0.5783
HbA1c (%)	6.90 (5.60–10.00)	6.80 (4.60–10.20)	0.1490
Total cholesterol (mg/dL)	155.57 (90.04–376.17)	154.89 (91.38–347.89)	0.9210
HDL cholesterol (mg/dL)	44.45 (23.12–79.18)	44.30 ± 9.24	0.3290
LDL cholesterol (mg/dL)	84.34 (31.20–270.60)	80.85 (36.57–221.68)	0.6143
Triglycerides (mg/dL)	154.35 (62.37–390.53)	153.13 (70.06–609.08)	0.6330
Uric acid (mg/dL)	5.36 (3.39–9.56)	6.10 ± 1.53	0.0032
C peptide (ng/mL)	2.78 (0.49–8.08)	3.51 (0.28–10.50)	0.0007
ALAT (U/l)	16.72 (2.32–80.94)	20.66 (5.31–92.79)	0.0151
ASAT (U/l)	19.11 (9.75–130.85)	22.14 (11.16–78.41)	0.0024
Direct bilirubin (mg/dL)	0.18 (0.07–0.49)	0.22 (0.07–0.9)	0.0004
GGT (U/l)	27.35 (4.02–313.66)	31.33 (4.97–338.18)	0.0906
Albumin (g/dL)	4.62 (4.19–5.35)	4.65 ± 0.24	0.8173
Haptoglobin (g/l)	1.81 ± 0.61	1.54 (0.37–3.47)	0.0006
Ferritin (ng/mL)	80.75 (6.41–543.00)	109.00 (8.79–811.0)	0.0228
Creatinine (mg/dL)	0.79 (0.46–1.35)	0.87 (0.54–1.98)	0.0025

**Table 2:** Bivariate associations of platelet specific parameters with the other hematological and biochemical parameters (PTC = plateletcrit; PDW = platelet distribution width; HTC = hematocrit; HGB = hemoglobin; MCV = mean corpuscular volume; MCH = mean corpuscular hemoglobin; MCHC = mean corpuscular hemoglobin concentration; ASAT = aspartate aminotransferase; ALAT = alanine aminotransferase; GGT = gamma-glutamyl transpeptidase; HOMA-IR = Homeostatic Model Assessment for Insulin Resistance; PIIINP = procollagen III N-terminal propeptide; HbA1c = glycated hemoglobin; FLI = Fatty Liver Index; *: *p* < 0.05; **: *p* < 0.01; ***p < 0.001; ****: *p* < 0.0001).

	Platelet Count r [95%CI]	PTC r [95%CI]	PDW r [95%CI]
Sex	−0.27 [−0.39; −0.15] ****	−0.30 [−0.41; −0.18] ****	0.19 [0.07; 0.30] **
Alcohol intake	−0.17 [−0.29; −0.05] **	−0.22 [−0.34; −0.10] ***	0.11 [−0.02; 0.23]
Red blood cells	0.03 [−0.09; 0.15]	−0.001 [−0.12; 0.12]	0.22 [0.10; 0.34] ***
HTC	−0.12 [−0.24; 0.003] *	−0.11 [−0.23; 0.01]	0.26 [0.15; 0.37] ****
HGB	−0.11 [−0.22; 0.01]	−0.17 [−0.29; −0.05] ***	0.27 [0.15; 0.38] ****
MCV	−0.21 [−0.32; −0.09) ***	−0.22 [−0.34; −0.10] ***	−0.02 [−0.14; 0.10]
MCH	−0.25 [−0.36; −0.13] ****	−0.31 [−0.42; −0.19] ****	0.01 [−0.11; 0.13]
MCHC	−0.22 [−0.33; −0.10] ***	−0.32 [0.43; −0.20] ****	0.11 [−0.01; 0.23]
Leucocytes	0.44 [0.33; 0.53] ****	0.52 [0.43; 0.61] ****	−0.07 [−0.19; 0.05]
Neutrophils	0.39 [0.28; 0.49] ****	0.47 [0.36; 0.56] ****	−0.05 [−0.17; 0.07]
Lymphocytes	0.26 [0.14; 0.37] ****	0.35 [0.24; 0.46] ****	−0.09 [−0.21; 0.03]
Monocytes	0.18 [0.06; 0.30] **	0.23 [0.11; 0.34] ***	−0.04 [−0.17;0.08]
Ferritin	−0.18 [−0.30; −0.06] **	−0.20 [−0.32; −0.08] ***	0.13 [0.003; 0.25] *
Haptoglobin	0.26 [0.15; 0.37] ****	0.30 [0.18; 0.41] ****	−0.10 [−0.22; 0.03]
PIIINP	0.21 [0.09; 0.33] ***	0.21 [0.09; 0.33] ***	−0.12 [−0.24; 0.001] *
HOMA-IR	−0.20 [−0.31; −0.07] **	−0.18 [−0.29; −0.06] **	0.20 [0.08; 0.32] ***
Blood glucose	0.006 [−0.12; 0.13]	−0.03 [−0.15; 0.10]	0.16 [0.04; 0.28] **
HbA1c	0.08 [−0.04; 0.21]	0.06 [−0.06; 0.19]	0.12 [−0.004; 0.24]
Triglycerides	−0.01 [−0.13; 0.11]	−0.03 [−0.15; 0.09]	0.15 [0.03; 0.27] *
HDL cholesterol	0.11 [−0.01; 0.23]	0.14 [0.01; 0.26] *	−0.14 [−0.26; −0.02] *
Creatinine	0.18 [−0.30; −0.06] **	−0.20 [−0.31; −0.08] **	0.17 [0.05; 0.29] **
ALAT	−0.18 [−0.30; −0.06] **	−0.18 [−0.30; −0.06] **	0.13 [0.005; 0.25] *
ASAT	−0.26 [−0.37; −0.14] ****	−0.27 [−0.38; −0.16] ****	0.15 [0.03; 0.27] *
GGT	−0.15 [−0.27; −0.03] *	−0.20 [−0.31; −0.08] **	0.19 [0.07; 0.30] **
Direct bilirubin	−0.26 [−0.37; −0.14] ****	−0.28 [−0.39; −0.16] ****	0.06 [−0.06; 0.18]
FLI	−0.15 [−0.27; −0.03] *	−0.11 [−0.23; 0.01]	0.19 [0.07; 0.31] **

**Table 3:** The multiple regression analyses of correlation between main platelet indices and other clinical and laboratory parameters (PTC = plateletcrit; PDW = platelet distribution width; MCH = mean corpuscular hemoglobin; HOMA-IR = Homeostatic Model Assessment for Insulin Resistance; GGT = gamma-glutamyl transpeptidase; PIIINP = procollagen III N-terminal propeptide; *: *p* < 0.05; **: *p* < 0.01; ***p < 0.001; ****: *p* < 0.0001).

	Platelet Count	PTC	PDW
	R^2^ = 33.39%; *p* < 0.0001	R^2^ = 43.20%; *p* < 0.0001	R^2^ = 16.25%; *p* < 0.0001
	β [SE]; t Ratio	β [SE]; t Ratio	β [SE]; t Ratio
Sex	−23.19 [9.01]; 2.57 *	−0.029 [0.008]; 3.52 ***	0.157 [0.055]; 2.83 **
Alcohol intake	0.10 [0.77]; 1.30	1.506 × 10^−5^ [0.001]; 0.02	−0.011 [0.005]; 2.23 *
Leucocytes	14.35 [2.03]; 7.07 ****	0.018 [0.002]; 9.63 ****	−0.017 [0.012]; 1.35
MCH	−3.11 [1.82]; 1.71	−0.003 [0.002]; 1.68	0.0002 [0.011]; 0.02
HOMA-IR	−10.34 [2.70]; 3.82 **	−0.011 [0.002]; 4.29 ****	0.060 [0.017]; 3.58 ***
Haptoglobin	6.10 [6.50]; 0.94	0.002 [0.006]; 0.30	−0.032 [0.040]; 0.81
Ferritin	0.02 [0.03]; 0.55	2.792 × 10^−5^ [0.000]; 0.96	−0.0003 [0.0002]; 1.42
ASAT	−0.61 [0.47]; 1.31	0.0006 [0.0004]; 1.49	−0.001 [0.003]; 0.23
ALAT	0.33 [0.40]; 0.84	−0.0005 [0.0004]; 1.37	0.0002 [0.002]; 0.09
Direct bilirubin	−93.12 [37.68]; 2.47 *	−0.079 [0.035]; 2.26 *	0.274 [0.232]; 1.18
GGT	0.02 [0.01]; 0.25	−4.479 × 10^−6^ [0.000]; 0.05	0.001 [0.001]; 2.38 *
Creatinine	−10.16 [17.75]; 0.57	−0.0002 [0.016]; 0.01	0.039 [0.109]; 0.36
PIIINP	0.13 [0.11]; 1.10	0.0001 [0.0001]; 1.25	−0.0004 [0.001]; 0.57

## Data Availability

Data may be available upon request.
